# Supplementary figures and images for: The relationship between postpartum depression and appropriate infant feeding practice in eastern zone of Tigray, Ethiopia: A comparative cross-sectional study
Source: PLoS One. 2023 Jan 25;18(1):e0280141. doi: 10.1371/journal.pone.0280141 (PMC9876352; doi:10.1371/journal.pone.0280141)

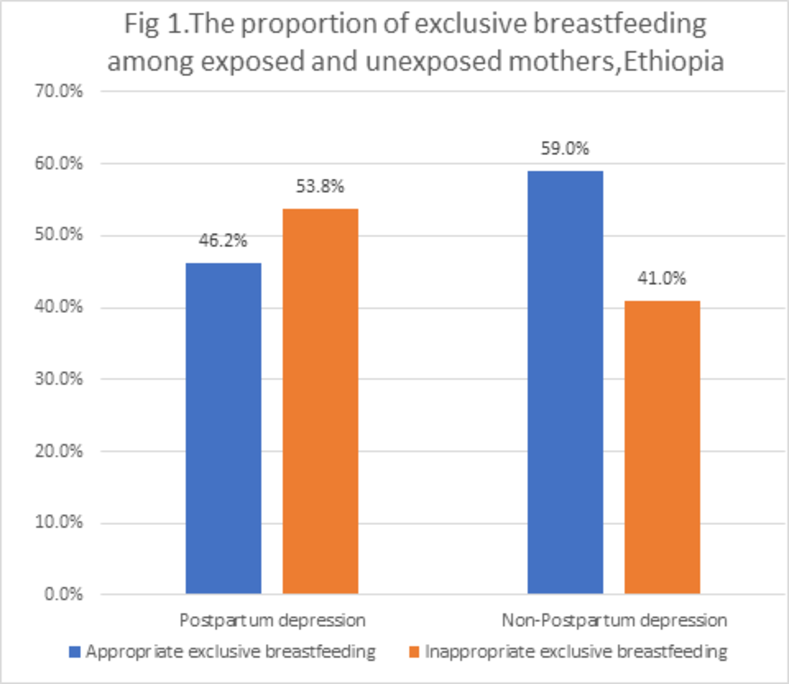

Supplement: S1 Fig — (TIF) [file pone.0280141.s001.tif]

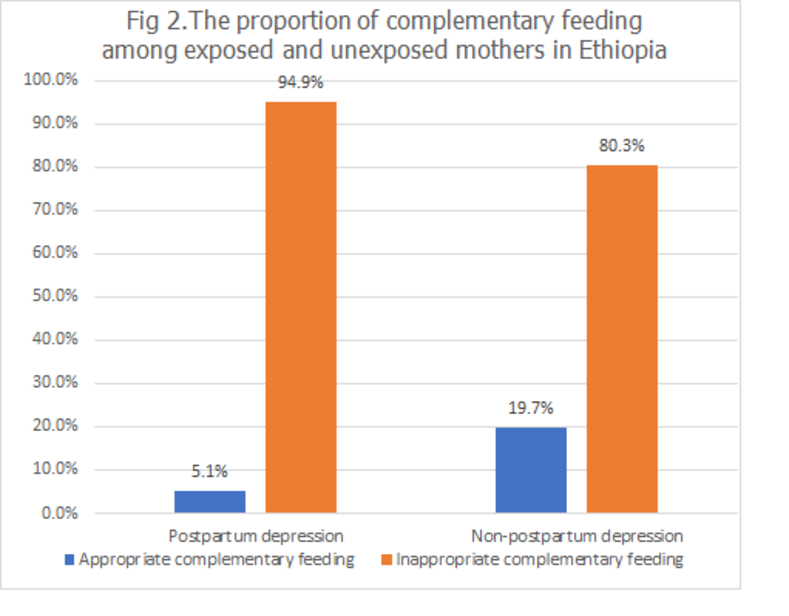

Supplement: S2 Fig — (TIF) [file pone.0280141.s002.tif]
