# Supplementary material for: The relationship between postpartum depression and appropriate infant feeding practice in eastern zone of Tigray, Ethiopia: A comparative cross-sectional study
Source: PLoS One. 2023 Jan 25;18(1):e0280141. doi: 10.1371/journal.pone.0280141 (PMC9876352; doi:10.1371/journal.pone.0280141)
Supplement: S1 Questionnaire — (DOCX) [file pone.0280141.s004.docx]

**English Questionnaire**

Participant ID No: ______ Date of interview: ____/___/_____ Kebeles: ______
Starting time: _____________Ending time: ___________

Please encircle the correct response which to indicate number and write a correct number example in Q101 Maternal age in years, or infant age in days

Part I: Socio-demographic characteristics

| **S/No** | **Questions** | **Responses** | **Code** | **Skip** | |
| --- | --- | --- | --- | --- | --- |
| 101 | Maternal age | _____ years |  |  | |
| 102 | What is your marital status? | Married  Single  Divorced  Separated  Widowed | 1  2  3  4  5 |  | |
| 103 | What is your educational status? | Illiterate  Only read and write  Primary school (grade 1-8)  Secondary school (grade 9-12)  College and above (> Grade 12) | 1  2  3  4  5 |  | |
| 104 | What is your occupation? (More than one answer is possible). | Housewife  Farmer  Merchant  Government employed  Student  Other (specify) ________ | 1  2  3  4  5  98 |  | |
| 105 | What is your monthly family income? | _______ in Ethiopian birr (ETB) |  |  | |
| 106 | Where is your Residence? | Urban  Rural | 1  2 |  | |
| 107 | Religion | Orthodox  Muslim  Protestant  Catholic  Other (specify) ________ | 1  2  3  4  98 |  | |
| 108 | Did you partner uses any substance like chat, smoking or any other drug? | YES  NO | 1  2 | |  |
| 109 | Infant age (in days) | _____ days |  |  | |
| 110 | Infant sex | Male  Female | 1  2 |  | |
| 111 | Birth interval (in months) | ________ |  |  | |
| 112 | Birth order of the child | _________ |  |  | |

Part II: Health care related characteristics

| 201 | Parity | ________ |  |  |
| --- | --- | --- | --- | --- |
| 202 | Where did you give birth [Name]? | Home  Health facility | 1  2 |  |
| 203 | Mode of delivery | Normal delivery  Caesarian section | 1  2 |  |
| 204 | When you got pregnant with [Name], did you want to get pregnant at that time? | YES  NO | 1  2 |  |
| 205 | Did you see any one for antenatal care for this pregnancy? | YES  NO | 1  2 | If no skip  Q206 |
| 206 | If the answer to Q116 is Yes, how many times did you receive ANC for this pregnancy? | Once  Twice  Three times  Four times | 1  2  3  4 |  |
| 207 | Did you receive any care after you deliver with [Name]? | YES  NO | 1  2 |  |

Part III: Self-reporting Questionnaire (SRQ-20)

Study participant ID __________

As you have recently had a baby, we would like to know how you are feeling. The following questions are related to certain somatic and emotional symptoms that might bother you in the last 30 days. If you think the questions applies to you Say YES and say **NO** if it doesn’t apply to you.

| **S/No** | **SRQ-20 Questions** | **Response** | **Code** |
| --- | --- | --- | --- |
| 301 | Do you often have headaches? | YES  NO | 1  2 |
| 302 | Is your appetite poor? | YES  NO | 1  2 |
| 303 | Do you sleep badly? | YES  NO | 1  2 |
| 304 | Are you easily frightened? | YES  NO | 1  2 |
| 305 | Do your hands shake? | YES  NO | 1  2 |
| 306 | Do you feel nervous, tense or worried? | YES  NO | 1  2 |
| 307 | Is your digestion poor? | YES  NO | 1  2 |
| 308 | Do you have trouble thinking clearly? | YES  NO | 1  2 |
| 309 | Do you feel unhappy? | YES  NO | 1  2 |
| 310 | Do you cry more than usual? | YES  NO | 1  2 |
| 311 | Do you find it difficult to enjoy your daily activities? | YES  NO | 1  2 |
| 312 | Do you find it difficult to make decisions? | YES  NO | 1  2 |
| 313 | Is your daily work suffering? | YES  NO | 1  2 |
| 314 | Are you unable to play a useful part in life? | YES  NO | 1  2 |
| 315 | Have you lost interest in things? | YES  NO | 1  2 |
| 316 | Do you feel that you are a worthless person? | YES  NO | 1  2 |
| 317 | Has the thought of ending your life been on your mind? | YES  NO | 1  2 |
| 318 | Do you feel tired all the time? | YES  NO | 1  2 |
| 319 | Are you easily tired? | YES  NO | 1  2 |
| 320 | Do you have uncomfortable feelings in your stomach? | YES  NO | 1  2 |
| **SRQ-20 Total Score** | | YES =  NO = | |

Part IVA: Infant feeding practice assessment tool

| **S/No** | **Questions** | **Responses** | **Code** | **Skip to** |
| --- | --- | --- | --- | --- |
| 401 | Date of interview | __/___/_____ dd/mm/yy) |  |  |
| 402 | Child’s date of birth | __/___/______dd/mm/yy) |  |  |
| 403 | Have you ever breastfed [Name]? | YES  NO | 1  2 | If ‘NO’ skip Q 303 |
| 404 | Since this time yesterday, have you breastfed your child [Name]? | YES  NO | 1  2 |  |
| 405 | Do you ever give your child anything to drink in a baby bottle? | YES  NO | 1  2 |  |
| 406 | How long after birth did you first put (NAME) to the breast for the first time?  If less than 1 hour, record ‘00' hours; if less than 24 hrs, record hrs; otherwise, record days? | Immediately______  Hours _____  Days______ |  |  |
| 407 | How many times did you breastfeed [NAME] last night between sunset and sunrise? | Times _ ___ |  |  |
| 408 | How many times did you breastfeed [NAME] yesterday during the daylight hours? | Times ______ |  |  |
| 409 | Since this time yesterday, has [NAME] received any of the following? |  |  |  |
|  | A) Vitamins, mineral supplements, medicine | YES  NO | 1  2 |  |
|  | B) Plain water | YES  NO | 1  2 |  |
|  | C)Sweetened or flavored water | YES  NO | 1  2 |  |

|  | D) Fruit juice | YES  NO | 1  2 |  |
| --- | --- | --- | --- | --- |
|  | E) Tea or infusions | YES  NO | 1  2 |  |
|  | F) Infant formula/bottle feeding | YES  NO | 1  2 |  |
|  | G) Tinned, powdered or fresh milk | YES  NO | 1  2 |  |
|  | H) Other liquids (Includes broths and clear soups) | YES  NO | 1  2 |  |
|  | I) Mushy or solid foods (Includes cereal, porridge, thick soups, or stews) | YES  NO | 1  2 |  |
|  | J) Oral Rehydration Salts (ORS) solution | YES  NO | 1  2 |  |

Part IV B: Complementary feeding (6-11months)

| 410 | | Did [Name] ever start eating any solid, semi-solid, or soft foods? Yesterday during the day or at night? | YES  NO | 1  2 |  |
| --- | --- | --- | --- | --- | --- |
| 411 | When did you start giving any solid, semi-solid, or soft foods [Name]? | Less than 6 months  At 6 months  Greater than 6 months | 1  2  3 |  |  |
| 412 | If Q309 is yes, What kind of solid, semi-solid, or soft foods did **(NAME)** eat?  Please describe everything that **(NAME)** ate yesterday during the day or night, whether at home or outside the home? |  |  |  |  |
|  | 1. Porridge, bread, rice, noodles, or other foods made from grains | YES  NO | 1  2 | |  |
|  | 1. Pumpkin, carrots, squash, or sweet potatoes that are yellow or orange inside | YES  NO | 1  2 | |  |
|  | 1. white potatoes, white yams, manioc, cassava, or any other foods made from roots | YES  NO | 1  2 | |  |
|  | 1. any dark green leafy vegetables | YES  NO | 1  2 | |  |
|  | 1. any other fruits or vegetables | YES  NO | 1  2 | |  |
|  | 1. liver, kidney, heart, or other organ meats | YES  NO | 1  2 | |  |
|  | 1. any meat, such as beef, pork, lamb, goat, chicken, or duck | YES  NO | 1  2 | |  |
|  | 1. any foods made from beans, peas, lentils, nuts, or seeds | YES  NO | 1  2 | |  |
|  | 1. cheese, yogurt, or other milk products | YES  NO | 1  2 | |  |
|  | 1. ripe mangoes, ripe papayas, | YES  NO | 1  2 | |  |
| 413 | How many times [Name] eat any of the food groups provided below in the last night? |  |  | |  |
|  | 1.grains, roots and tubers | Never  1-3 days  ≥4 | 0  1  2 | |  |
|  | 2.legumes and nuts | Never  1-3 days  ≥4 | 0  1  2 | |  |
|  | 3.dairy products (milk and yoghurt) | Never  1-3 days  ≥4 | 0  1  2 | |  |
|  | 4.flesh foods (meat, fish, poultry and meats) | Never  1-3 days  ≥4 | 0  1  2 | |  |
|  | 5.eggs | Never  1-3 days  ≥4 | 0  1  2 | |  |
|  | 6.vitamin A-rich fruits and vegetables | Never  1-3 days  ≥4 | 0  1  2 | |  |
|  | 7.other fruits and vegetables | Never  1-3 days  ≥4 | 0  1  2 | |  |

**ናይ ትግርኛ መሕተት**

ተሳታፊ ምስጢር ቁጽሪ ፡——— ቃለ-መሕትት ዝተገብረሉ ዕለት ፡ / / / ቀበሌ: .

ምሕታት ዝጀመረሉ ሰዓት：______________ምሕታት ዝወድኣሉ ሰዓት：_______

ነቲ ትኽክል መልሲ ዝተወሃበ ዝኮነ መማረጺ ክቢ ብምክባብ ይመልሱ።

ክፋል 1 .ማሕበራዊ ኩነታት ዝምልከት

| **ተ᎐ቁ** | **ሕቶ** | **መልሲ/መማረጽታት** | **ኮድ** | **ዝለል** | | |
| --- | --- | --- | --- | --- | --- | --- |
| 101 | ዕድመ አዶ（ብዓመት） | _____ ዓመት |  |  | | |
| 102 | ናይ ሓዳር ኩነታት ከመይ እዩ？ | ዝተመርዓወት  ዘይተመርዓወት  ተፋቲሐ  ተፈላሊና  ብዓል ቤታ ዝሞታ | 1  2  3  4  5 |  | | |
| 103 | ትምህርቲ ደረጅኣ ክንደይ እዩ？ | ዘይተምሃረት  ምጽሓፍን ምምባብን ጥራይ  ቀዳማይ ብርኪ ዝበጽሐት (ብርኪ 1-8)  ካልኣይ ብርኪ ዝበጽሐት (ብርኪ 9-12)  ኮሌጅ ወይ ካብኡ ንላዕሊ ዝበጽሐት | 1  2  3  4  5 |  | | |
| 104 | እንታይ ትሰርሒ ? (ካብ ሓደ ብላዕሊ መልሲ ይከአል). | ውሓለ ገዛ  ሓረስታይ  ነጋዳይ  መንግስቲ ሰራሕተኛ  ተማሃሪት  ካሊእ（ግለጽ）________ | 1  2  3  4  5  98 |  | | |
| 105 | ወርሓዊ ናይ ቤተ ሰብ እቶት ክንደይ እዩ? | ________ ብብር |  | | | |
| 106 | ትነብርሉ ከባቢ | ከተማ  ገጠር | 1  2 | |  | |
| 108 | ትክተልዮ ሃይማኖት？ | ኦርቶዶክስ  እስልምና  ካሊእ（ግለጽ）________ | 1  2  98 | | |  |
| 109 | ናይ ህጻን ዕድመ？(ብሙሉእ ብመዓልቲ) | _____ ብመዓልቲ |  | | | |
| 110 | ኣብ ናይ መጀመሪያ ቆልዓን ቀጺሉ ዘሎ ቆልዓን ዘሎ ዕድመ ኣፈላላይ？ | _________አዋርሕ |  | | | |
| 111 | እዚ ቆልዓ መበል ክንደየናይ እዩ？ | _________ |  | | | |

ክፋል 2 ：ጥዕና ኣዶ ዝተተሓዙ ሕቶታት

| 201 | በዝሒ በተሰብ ？ | ________ |  | |
| --- | --- | --- | --- | --- |
| 202 | ህጻን ዝወለድኪሉ ቦታ አበይ እዩ？ | ኣብ ገዛ  ኣብ ጥዕና ተቋም | 1  2 |  |
| 203 | እዚ ህጻን ዝወለድኪሉ ብምንታይ እዩ？ | ብማህጸን  ብከብደይ（ኦፕሬሽን） | 1  2 |  |
| 204 | እቲ ሕርሲ ብድሌት ናይ ክልቴኩም ድዩ ነይሩ？ | እወ  ኣይፋሉን | 1  2 |  |
| 205 | ኣብ እዋን ሕርሲ እዚ ቆልዓ እንዳሃለውኪ ቅድሚ ወሊድ ክትትል ጌርኪ ነኤርኪ ዶ？ | እወ  ኣይፋሉን | 1  2 | መልሲ አይፋሉን ተኮይኑ ናብ 207 ይሕለፉ |
| 206 | ንክንደይ ጊዜ ዝከውን ክትትል ጌርኪ ነኤርኪ？ | ሓደጊዜ  ክልተጊዜ  ሰለስተ ጊዜ  ኣርባዕተ ጊዜ | 1  2  3  4 |  |
| 207 | ድሕሪ ወሊድ ክትትል ጌርኪ ነኤርኪ ዶ？ | እወ  ኣይፋሉን | 1  2 | |

ክፋል 3 : Self-reporting Questionnaire (SRQ-20)

ተሳታፊ ቁጽሪ____________________ ዝተወድአሉ ዕለት_________________

ድሕሪ እዚ/እታ ቆልዓ ምስ ወለድኪ ዘሎ ስምዒት ንምፍላጥ።እዞም ዝስዕቡ ሕቶታት ምስ አእምሮኣዊ ስምዒታት ዝተትሓሓዙ እንትኮኑ ኣብዝሓልፉ 30 መዓልትታት ዘጨንቁኺ ኢሉውን ዘተሓሳስቡኺ ሕቶታት ዝሓቆፈ እዩ።እንድሕር እቲ ሕቶ ዝውክለኪ እንተኮይኑ **እወ** ብምባል ዘይውክለኪ እንተኮይኑ ድማ **ኣይፋሉን** ብምባል ይመልሱ። .

| **ተ᎐ቁ** | **SRQ-20 ሕቶታት** | **መልሲ** | **ኮድ** |
| --- | --- | --- | --- |
| 301 | መብዛሕቲኡ ጊዜ ድኻም ይስማዓኪ ዶ？ | እወ  ኣይፋሉን | 1  2 |
| 302 | ካብ ዝሓለፈ ወርሒ ጀሚሩ ናይ ምግቢ ድሌትኪ ቀኒሱ ዶ？ | እወ  ኣይፋሉን | 1  2 |
| 303 | ድቃስ ቡዙሕ ጊዜ ይአብየኪዶ？ | እወ  ኣይፋሉን | 1  2 |
| 304 | ብቀሊሉ ፍርሕ ፍርሕ ትብሊ ዶ ？ | እወ  ኣይፋሉን | 1  2 |
| 305 | ኣእዳውኪ ይንቅጥቀጥ ዶ？ | እወ  ኣይፋሉን | 1  2 |
| 306 | ውጥረት ፣ጭንቀት ይስመዐኪ ዶ？ | እወ  ኣይፋሉን | 1  2 |
| 307 | ምግቢ ቶሎ ምሕቃቅ ይአብየኪ ዶ？ | እወ  ኣይፋሉን | 1  2 |
| 308 | ብግልጺ ናይ ምሕሳብ ችግር የጋጥመኪ ዶ？ | እወ  ኣይፋሉን | 1  2 |
| 309 | ካብ ዝሓለፈ ወርሒ ጀሚሩ ሕጉስቲ ዘይሙካን ስሚዒት ይስመዓኪ ዶ？ | እወ  ኣይፋሉን | 1  2 |
| 310 | ብዙሕ ጊዜ ካብ ዓቀን ንላዕሊ ትነብዒ ዶ？ | እወ  ኣይፋሉን | 1  2 |
| 311 | አብ መዓልታዊ ትሰርሕዮ ስራሕ ብቀሊሉ ዘይምሕጓስ ስሚዒት ይስመዓኪ ዶ | እወ  ኣይፋሉን | 1  2 |
| 312 | ካብ ዝሓለፈ ወርሒ ጀሚሩ ውሳኔ አብ ምዉሳን ትጽገሚ ዶ？ | እወ  ኣይፋሉን | 1  2 |
| 313 | ማዓልታዊ ስራሕ ንምስራሕ ትጽገሚ ዶ？ | እወ  ኣይፋሉን | 1  2 |
| 314 | ኣብ እዋን ፉሉይ ሓጎስ ዘይምሕጓስ ？ | እወ  ኣይፋሉን | 1  2 |
| 315 | ካብ ዝሓለፈ ወርሒ ጀሚሩ አብ ነገራት ድሌት ዘይም | እወ  ኣይፋሉን | 1  2 |
| 316 | ዋጋ የብለይን（አይረብሕን） እየ ኢልኪ ትሓስቢ ዶ？ | እወ  ኣይፋሉን | 1  2 |
| 317 | ዓርሰ ቅትለት ንምፍጻም ሃሲብኪ ትፈልጢ ዶ？ | እወ  ኣይፋሉን | 1  2 |
| 318 | ኩሉ ጊዜ ድኻም ይስማዓኪ ዶ？ | እወ  ኣይፋሉን | 1  2 |
| 319 | ብቀሊሉ ትደኽሚ ዶ？ | እወ  ኣይፋሉን | 1  2 |
| 320 | አብ ጨጎራኺ ቃንዛ ይስመዓኪዶ？ | እወ  ኣይፋሉን | 1  2 |
|  | SRQ-20 ውጽኢት ድምር= | ጠቅላላ እወ=  ጠቅላላ ኣይፋሉን= | |

**ክፋል 4 A : ናይ ትሕቲ ሓደ ዓመት ኣመጋግባ ስርዓት መለክዒ**

| ተ᎐ቁ | ሕቶ | መልሲ | ኮድ | ዝለል | |
| --- | --- | --- | --- | --- | --- |
| 401 | ቃለ-መሕትት ዝተገብረሉ ዕለት | ______/______/________መ/ወ/ዓ |  |  | |
| 402 | ህጻኑ ዝተወለደሉ ዕለት | ______/______/________መ/ወ/ዓ |  |  | |
| 403 | ነዚ ቆልዓ እስካብ ሀዚ ኣጥቢብኪዮ ትፈልጢ ዶ？ | እወ  ኣይፋሉን | 1  2 |  | |
| 404 | ነዚ ህጻን ካብ ጸባ ኣዶ ወጻኢ ብጡጦ ሂብኪዮ ትፈልጢ ዶ？ | እወ  ኣይፋሉን | 1  2 |  | |
| 405 | እዚ ህጻን ምስ ወለድኪ ድሕሪ ክንደይ ጡብ ምጥቧብ ጀሚርኪዮ？ቅድሚ ሓደ ሰዓት እንተ ኾይኑ 00 ሰዓት ትሕቲ 24 ሰዓት እንተኮይኑ ብ ሰዓታት እንተዘይ ኮይኑ ድማ ብመዓልትታት ኣቀምጥ | ______ ወድያውኑ  ______ ሰዓታት  ______ መዓልትታት |  |  | |
| 406 | ካብ ጸሓይ ዓራርቦ ክሳብ ወጋሕታ ዘሎ ንክንደይ ሰዓት ኣጥቢብኪዮ/ያ？ | ______ ጊዜ |  |  | |
| 407 | ኣብ እዋን ቀትሪ ንክንደይ ሰዓት ኣጥቢብኪዮ/ያ？ | ______ ጊዜ |  |  | |
| 408 | ካብ ትማሊ ክሳብ ሐዚ ካብዞም ዝተዘርዘሩ ወሲዱ/ዳ ነይሩ/ራ ዶ? |  |  |  | |
|  | ሀ) ቫይታሚን፣ሚነራል ወይ ከዓ መድሓኒት | እወ  ኣይፋሉን | 1  2 | |  |
|  | ለ) ናይ ቡንቧ ማይ | እወ  ኣይፋሉን | 1  2 | |  |
|  | ሐ)ሽኮራዊ መስተ | እወ  ኣይፋሉን | 1  2 | |  |
|  | መ) ጽሟቅ ናይ ፍረምረ | እወ  ኣይፋሉን | 1  2 | |  |
|  | ረ) ሻሂ | እወ  ኣይፋሉን | 1  2 | |  |
|  | ሰ) ፎርሙላ ጸባ | እወ  ኣይፋሉን | 1  2 | |  |
|  | ሸ) ዕሹግ ጸባ | እወ  ኣይፋሉን | 1  2 | |  |
|  | ቀ) ካልኦት ፈሰስቲ ከም በዓል | እወ  ኣይፋሉን | 1  2 | |  |
|  | በ) ዝተጥሓነ ድንሽ፣ገዓት፣ ሓፊስ ጸብሒ | እወ  ኣይፋሉን | 1  2 | |  |
|  | ተ) ጨው ኢንግሊዝ(ORS) | እወ  ኣይፋሉን | 1  2 | |  |

ክፋል 4 B:ተወሳኪ ምግቢ ዝምልከቱ ሕቶታት (6-11 ወርሒ)

| 409 | ተወሳኺ ምግቢ(ካብ ጸባ ወጻ)ኢ ጀሚርክሉ ዶ？ | እወ  ኣይፋሉን | 1  2 |
| --- | --- | --- | --- |
| 410 | መዓዝ እዩ ን[ስም ]ተወሳኺ ምግቢ ዝጀመርክሉ？ | ቅድሚ ሽድሽተ ወርሒ  ኣብ ሽድሽተ ወርሒ  ድሕሪ ሽድሽተ ወርሒ | 1  2  3 |
| 411 | ኣብ ዝሓለፈ መዓልቲ [ስም] ካብዞም ዝተዘርዘሩ ምግብታት ወሲዱ/ዳ ነይሩ/ራ ዶ？ |  |  |
|  | ሀ)ገዓት፣ባኒ፣ሩዝ ወይከዓ ካልኦት ምግብታት ካብ ኣዝርእቲ ዝተዳለዉ | እወ  ኣይፋሉን | 1  2 |
|  | ሀ)ካሮት፣ሽኮር ድንሽ፣ዱባ፣ፓስታ | እወ  ኣይፋሉን | 1  2 |
|  | ለ)ድንሽ ወይ ከዓ ካብ ሱሮም ዝብላዕ ምግብታት ዝተዳለወ | እወ  ኣይፋሉን | 1  2 |
|  | ሐ)ቆጽለ መጽሊ ዝብልዑ ምግብታት ከም በዓል ቆስጣ፣ሰላጣ ወይ ከዓ ሓምሊ | እወ  ኣይፋሉን | 1  2 |
|  | መ)ፍረምረታት | እወ  ኣይፋሉን | 1  2 |
|  | ረ)ጸላም ከብዲ፣ልቢ ፣ኩላሊት ናይ እንስሳ | እወ  ኣይፋሉን | 1  2 |
|  | ሰ)ናይ ብዕራይ ስጋ፣ጤል ስጋ፣በጊዕ ስጋ ፣ዓሳ ስጋ ወይ ድማ ናይ ደርሆ ስጋ | እወ  ኣይፋሉን | 1  2 |
|  | ሸ)ካብ ጥራምረ ዝዳለዉ ምግብታት ከም ዓተር ፣ዓይኒ ዓተር ፣ፉል ወይ ድማ ቧልደንጓ | እወ  ኣይፋሉን | 1  2 |
|  | ቀ)ኣጅቦ፣ርግኦ፣ ጠስሚ፣ ጮማን ውጽኢት ጸባ | እወ  ኣይፋሉን | 1  2 |
|  | በ)ሽኮር ዘለዎም ምግብታት ከም ቸኮላታ፣ካሪመለ፣ፓስቲ፣ኬክ ወይ ድማ ብሽኩቲ | እወ  ኣይፋሉን | 1  2 |
|  | ተ)ተወሰኽቲ መመቀርቲ ምግብታት ከ ቃሪያ፣ቅመማ ቅመም | እወ  ኣይፋሉን | 1  2 |
|  | ተ)ከም ማንጎ፣ፓፓዮ ኣራንሺ ብ ቫይታሚን A ዝበልጸጉ ፍረምረታት | እወ  ኣይፋሉን | 1  2 |
| 412 | ኣብ ዝሓለፈ ሸውዓተ መዓልቲ ውሽጢ ንክንደይ ጊዜ ዝኣክል ካብዞም ዝተዘርዘሩ ጉጅለ ምግብታት ወሲዱ? |  |  |
|  | ሀ)ኣዝርቲ ፣ሱሮም ዝብላዕ | ጊዜ |  |
|  | ለ) ጥረምረ | ጊዜ |  |
|  | ሐ)ጸባን ውጽኢት ጸባን(ጸባ፣ርግኦን ኣጅቦ) | ጊዜ |  |
|  | መ)ስጋ ናይ(ዓሳ፣ክፍሊ ኣካላት እንስሳ ክም በዓል ጸላም ከብዲንኩላሊት) | ጊዜ |  |
|  | ረ)እንቋቁሖታት | ጊዜ |  |
|  | ሰ)ብ ቫይታሚን A ዝበልጸጉ ፍረምረታትን ኣቁጽልትን | ጊዜ |  |
|  | ሸ)ካልኦት ፍረምረታትን ኣቁጽልትን | ጊዜ |  |
