# Supplementary material for: The relationship between postpartum depression and appropriate infant feeding practice in eastern zone of Tigray, Ethiopia: A comparative cross-sectional study
Source: PLoS One. 2023 Jan 25;18(1):e0280141. doi: 10.1371/journal.pone.0280141 (PMC9876352; doi:10.1371/journal.pone.0280141)
Supplement: Attachment — Submitted filename: Responses to Reviewers.docx [file pone.0280141.s005.docx]

Dear Editors,

We greatly appreciate the time and effort put forth by reviewers and editors to improve our paper, ‘‘The relationship between postpartum depression and appropriate infant feeding practice in Eastern zone of Tigray, Ethiopia: A comparative cross-sectional study” which we have addressed below. If any responses are unclear or you wish additional changes, please let us know.

Editor comments

Comment 1: Please ensure that your manuscript meets PLOS ONE's style requirements, including those for file naming.

Author Response**:** The manuscript has been revised intensively in line with the journal’s requirements. Therefore, the title has changed to sentence case (Page 1) and asterixis symbol used in corresponding author (line 15 of page 1) and added a city after the institution in the affiliation part (line 10 of page 1).

Comment 2**:** Please include your tables as part of your main manuscript and remove the individual files. Please note that supplementary tables should be uploaded as separate "supporting information" files.

Author Response**:** We have also modified the figure cite right next to the paragraph represented and removed from main manuscript (line 253-254 of page 15) and uploaded as separate file.

Comment 3. Please provide additional details regarding participant consent. In the ethics statement in the Methods and online submission information, please ensure that you have specified whether consent was written or verbal/oral. If consent was verbal/oral, please specify:

1) whether the ethics committee approved the verbal/oral consent procedure*,*

2) why written consent could not be obtained, and

3) how verbal/oral consent was recorded. If your study included minors, please state whether you obtained consent from parents or guardians in these cases. If the need for consent was waived by the ethics committee, please include this information

Author Response: We have agreed and incorporated additional details about participants consent.

1) Informed verbal consent was obtained after approval by the research ethical committee number (REC/0018/2019).

2) Since national and regional statistics show the nation’s (Ethiopia) rural and semi urban areas which account the large proportion of geographical area of the country have high rate of illiteracy that makes difficult to obtain written consent from participants. In addition, our study neither involve with sensitive issue nor uses any procedure to adversely affect participants welfare.

3) After reading the whole information about the purpose, risk, beneficence and non-maleficence, participants were asked whether they are willing to participate in the study with a statement

“Do you give your permission for me to interview you?”

A) Yes, I give you my permission

B) No, I am not willing to participate

If he/she agrees to participate we tick on the box.

informed verbal consent was recorded However, we believe that the minors (infants in our case) are not directly involved in the study only the mothers provide the information by remembering the past feeding practice.

Comment 4: Thank you for stating the following financial disclosure: "The funders had no role in study design, data collection and analysis, decision to publish, or preparation of the manuscript" At this time, please address the following queries:

A) Please clarify the sources of funding (financial or material support) for your study. List the grants or organizations that supported your study, including funding received from your institution.

Author response: The funding source of this was Addis Ababa University. Amount received 25,000 Ethiopian Birr.

B) State what role the funders took in the study. If the funders had no role in your study, please state: “The funders had no role in study design, data collection and analysis, decision to publish, or preparation of the manuscript.”

Author response: “The funders had no role in study design, data collection and analysis, decision to publish, and preparation of the manuscript.”

C) If any authors received a salary from any of your funders, please state which authors and which funders. Please include your amended statements within your cover letter; we will change the online submission form on your behalf.

Comment 5. We note that you have stated that you will provide repository information for your data at acceptance. Should your manuscript be accepted for publication, we will hold it until you provide the relevant accession numbers or DOIs necessary to access your data. If you wish to make changes to your Data Availability statement, please describe these changes in your cover letter and we will update your Data Availability statement to reflect the information you provide.

Author response**:** Thank you for the suggestion. I will make the necessary data available as per your request.

Comment 6**.** PLOS requires an ORCID iD for the corresponding author in Editorial Manager on papers submitted after December 6th, 2016. Please ensure that you have an ORCID iD and that it is validated in Editorial Manager.

Author response: I do have ORCID iD and I already validated in PLOS editorial manager.

Comment 7: Please ensure that you refer to Figure 1 in your text as, if accepted, production will need this reference to link the reader to the figure.

Author response: I have corrected the figure 1 cite to Fig 1 and put it in separate file (line 253 of page 15)

Comment 8. We note you have included a table to which you do not refer in the text of your manuscript. Please ensure that you refer to Tables 1-3 in your text; if accepted, production will need this reference to link the reader to the Tables

Author response: Thank you for the comment. we have corrected it accordingly.

[Note: HTML markup is below. Please do not edit.]

Reviewers' comments:

Reviewer's Responses to Questions

Comments to the Author

1. Is the manuscript technically sound, and do the data support the conclusions?

The manuscript must describe a technically sound piece of scientific research with data that supports the conclusions. Experiments must have been conducted rigorously, with appropriate controls, replication, and sample sizes. The conclusions must be drawn appropriately based on the data presented.

Reviewer #1: Yes

Reviewer #2: Yes

2. Has the statistical analysis been performed appropriately and rigorously?

Reviewer #1: Yes

Reviewer #2: No

3. Have the authors made all data underlying the findings in their manuscript fully available?

The PLOS Data policy requires authors to make all data underlying the findings described in their manuscript fully available without restriction, with rare exception (please refer to the Data Availability Statement in the manuscript PDF file). The data should be provided as part of the manuscript or its supporting information, or deposited to a public repository. For example, in addition to summary statistics, the data points behind means, medians and variance measures should be available. If there are restrictions on publicly sharing data—e.g. participant privacy or use of data from a third party—those must be specified.

Reviewer #1: Yes

Reviewer #2: Yes

4. Is the manuscript presented in an intelligible fashion and written in standard English?

PLOS ONE does not copyedit accepted manuscripts, so the language in submitted articles must be clear, correct, and unambiguous. Any typographical or grammatical errors should be corrected at revision, so please note any specific errors here.

Reviewer #1: No

Reviewer #2: Yes

5. Review Comments to the Author

Please use the space provided to explain your answers to the questions above. You may also include additional comments for the author, including concerns about dual publication, research ethics, or publication ethics. (Please upload your review as an attachment if it exceeds 20,000 characters)

**Reviewers' comments**:

Reviewer #1: Thank you for addressing an important topic. It is great to highlight the impact of PPD on infant feeding practices and such articles should also highlight the need to reduce the stigma associated with mental health. It would be good to add a few sentences on some background on the extent of Postpartum depression globally and the extent of underreporting. In addition, would be good to highlight the impact on women themselves.

Author response: we greatly appreciate for the compliment and constructive comments. We have included global burden of Postpartum depression and the effect of Postpartum depression on mothers and infants on background**.**

In the abstract, within the conclusion the authors write -"this study showed low prevalence and postpartum depression"- it is not clear what has low prevalence. Please state where PPD is linked with poor feeding practices. What about other children in the family- do they have a similar fate?

Author response: thank you for your view, it has been revised and stated in conclusion part as “This study showed low prevalence of appropriate infant feeding practice”. PPD is statistically linked with reduction of appropriate feeding practice.

Yes, we believe children in the same family will have similar fate because the unrecognized and untreated PPD results in reduced functioning of mother emotionally and physically which in turn have a negative influence of mother-infant interaction. Moreover, the persistent effect of untreated PPD will account for inability to take care of children in the family.

Please break your sentences and add the appropriate source according for the sentence. In lines 56-61, it is unclear which source is relevant for what statistics you are quoting.

Author response: Thank you for the view, we break the sentences and cited appropriately it is revised as follows “…….young child feeding practice is attributed to 45% of all child deaths in which two thirds of those deaths occurred in the first year of life [2]”

The description of the mothers in lines 195-205 could focus on bringing out the differences between the mothers who had PPD and those who did not. The description is hard to go through. Maybe better to summarize the similarities and focus more on the differences.

Author response: Thank you for your suggestion, we have tried to focus on variables which shows significant difference using Chi square and factors that are believed to provide image about study participants to the reader were summarized.

Is there a linkage between any history of depression in previous pregnancies and PPD in future?

Author response: Yes, there is an association between any history of depression in previous pregnancies and PPD but sometimes can occur independently.

Is that important and was that asked?

Author response: Thank you for your great question, Not asked because most literatures has been suggested that many of the postpartum mood disorders may actually begin during pregnancy or that antepartum depression increases the risk of developing PPD. It’s inevitable for a woman with antenatal depression/anxiety to have PPD and vice versa, mothers with PPD had experience antenatal depression/ anxiety. The other issue for not asking is we made decision not to introduce bias (recall bias while asking previous history of depression)

Check line 211. Incomplete sentence?

Author response: revised.

The discussion on birth order is not clearly stated. Please revise lines 287-295 to more clearly articulate your findings first.

The authors can strengthen the policy and program recommendations for PPD and its impact. Early recognition, raising awareness of symptoms associated with PD, eliminating stigma, involving the extended household and community to support women going through PPD so infant feeding practices are not impacted are some considerations.

A strong edit of the entire article is needed.

Reviewer #2: I found this article very interesting, but some changes should be made. There are some typo errors, specially missing spaces in text. I find the terms "exposed and unexposed" confusing and would advise to change them.

Author response: we have revised it intensively and correct all suggested typo error in the manuscript and changed exposed into mothers with PPD and unexposed changed mothers without PPD throughout the paper.

Abstract:

-rewrite the first sentence of the conclusion for clarity "this study showed low prevalence and postpartum depression as an important contributor to the appropriate infant feeding practice".

Author response: Thank you for the view, we have revised and stated in conclusion as follows “……This study showed low prevalence of appropriate infant feeding practice…”

Introduction:

- line 76: there is a typo, with a point before the word "studies" that should be corrected: "Ethiopia [9,10] .Studies"

Author response: revised and corrected.

Statistics:

-Control variables must be specified

Author response: The control variable are mothers without PPD.

Results:

-line 189 typo error, capital letter that should not be there in "retrieved Complete data".

Author response: revised and corrected.

- the sociodemographic variables results should be compared between groups through statistic tests (chi squared, T test...) to assess if statistically significant differences arise. This information should be included in the final tables, and it should be decided which data to include in the text and which in the table, so the text would be more easily read.

Author response: Revised and corrected

- it would be very important to describe, if possible, why the feeding was not appropriate, e.g. use of other foods before 6 months, interruption of breastfeeding, lack of variety in the diet, etc. This could help to have a better understanding of the reasons why an appropriate feeding practice do not happen (and what measures could be implemented to improve this practice).

Author response: Thank you for your suggestion. The appropriateness of feeding practice depends on age category of infant, mothers/caregiver with PPD give plain water frequently to infants less than 6 months of age and it was statistically significant(P-value-0.021) this was the main reason for inappropriateness of feeding practice among < 6 month of age, and the second important reason was receiving other liquids such as soup and broth before the age of six month. For infants 6-11 months of age, the main reason for inappropriate feeding was difficulty to receive minimum acceptable diet which was statistically significant (P-value = 0.04) different between mothers with PPD and mothers without PPD.

Discussion:

-line 255: clarify this sentence

Author response: Sentence clarified

- line 269: rewrite this sentence

Author response: Corrected

- line 292: correct typo ".this"

Author response: Corrected

- have the authors considered that the most frequent depressive symptoms described (headache, being tired) could be related to the difficulties inherently tied to breastfeeding? Could it be therefore a confusion between the consequences of breastfeeding and depression?

Author response: We have tried to put this temporality issue as a limitation of the study and must be cautiously interpreted as it would not enough to determine the causal association.in a specific way, whether PPD causes feeding difficulties or having feeding difficulty causes the mother to have depressive symptoms is mere possible to attain with such studies. Hence, we recommend further studies to be well designed prospective study to see the association between PPD and feeding difficulties or vice versa.

- again, to know exactly what was the reason for inappropriate feeding of the infants would be of the utmost importance to provide an interpretation of the difficulties mothers and particularly the ones suffering from PPD face.

Author response: Thank you for your great view, mothers/caregiver with PPD give plain water frequently to infants less than 6 months of age and it was statistically significant (P-value-0.021) this was the main reason for inappropriateness of feeding practice among < 6 month of age, and the second important reason was receiving other liquids such as soup and broth before the age of six month. For infants 6-11 months of age, the main reason for inappropriate feeding was difficulty to receive minimum acceptable diet which was statistically significant (P-value = 0.04) different between mothers with PPD and mothers without PPD.

Tables: all abbreviations used in the table should be clarified above.

Author response: revised and corrected. we have word abbreviated as Gov’t in to government in the table
