# Supplementary material for: The relationship between postpartum depression and appropriate infant feeding practice in eastern zone of Tigray, Ethiopia: A comparative cross-sectional study
Source: PLoS One. 2023 Jan 25;18(1):e0280141. doi: 10.1371/journal.pone.0280141 (PMC9876352; doi:10.1371/journal.pone.0280141)
Supplement: Attachment — Submitted filename: Responses to Reviewers.docx [file pone.0280141.s006.docx]

Dear Editors,

We greatly appreciate the time and effort put forth by reviewers and editors to improve our paper, ‘‘The relationship between postpartum depression and appropriate infant feeding practice in Eastern zone of Tigray, Ethiopia: A comparative cross-sectional study”. We have revised and incorporated the changes on the manuscript to address all the insightful suggestions raised by the editors and the reviewers.

PONE-D-21-02467R1

THE RELATIONSHIP BETWEEN POSTPARTUM DEPRESSION AND APPROPRIATE INFANT FEEDING PRACTICE IN EASTERN ZONE OF TIGRAY, ETHIOPIA: A COMPARATIVE CROSS-SECTIONAL STUDY

PLOS ONE

Dear Dr. Weldu,

Thank you for submitting your manuscript to PLOS ONE. After careful consideration, we feel that it has merit but does not fully meet PLOS ONE’s publication criteria as it currently stands. Therefore, we invite you to submit a revised version of the manuscript that addresses the points raised during the review process. Please carefully review abstract and write up of conclusions so ensure clarity and alignment with results from study.

Please submit your revised manuscript by Mar 05 2022 11:59PM. If you will need more time than this to complete your revisions, please reply to this message or contact the journal office at plosone@plos.org. When you're ready to submit your revision, log on to https://www.editorialmanager.com/pone/ and select the 'Submissions Needing Revision' folder to locate your manuscript file.

Please include the following items when submitting your revised manuscript:

A rebuttal letter that responds to each point raised by the academic editor and reviewer(s). You should upload this letter as a separate file labeled 'Response to Reviewers'.

A marked-up copy of your manuscript that highlights changes made to the original version. You should upload this as a separate file labeled 'Revised Manuscript with Track Changes'.

An unmarked version of your revised paper without tracked changes. You should upload this as a separate file labeled 'Manuscript'.

If you would like to make changes to your financial disclosure, please include your updated statement in your cover letter. Guidelines for resubmitting your figure files are available below the reviewer comments at the end of this letter.

If applicable, we recommend that you deposit your laboratory protocols in protocols.io to enhance the reproducibility of your results. Protocols.io assigns your protocol its own identifier (DOI) so that it can be cited independently in the future. For instructions see: https://journals.plos.org/plosone/s/submission-guidelines#loc-laboratory-protocols. Additionally, PLOS ONE offers an option for publishing peer-reviewed Lab Protocol articles, which describe protocols hosted on protocols.io. Read more information on sharing protocols at https://plos.org/protocols?utm_medium=editorial-email&utm_source=authorletters&utm_campaign=protocols.

We look forward to receiving your revised manuscript.

Kind regards,

Melissa F. Young, Ph.D.

Academic Editor

PLOS ONE

**Editor’s comment**

**Comment 1:** Please carefully review abstract and write up of conclusions so ensure clarity and alignment with results from study.

**Author response:** Thank you for your interesting insight and suggestion. We have thoroughly reviewed our results, discussion and conclusion and the following statements were corrected.

On conclusion part of abstract, we rephrased the statement with more sensible and aligned sentences with the write up. Now the statements read as follows **“The overall prevalence of appropriate infant feeding practice in the study area was low. The prevalence of appropriate infant feeding practice was significantly different between mothers without postpartum depression and mothers with postpartum depression”**.

**Line 43:** the word “**in addition”** to make the flow of sentences easily understandable.

**line 44**: The word **“predictors”** changed in to **“determinants”** to remove the repetition of words as recommended by the reviewer.

In addition, the original sentence provided in the first version of the manuscript has been rephrased by adding the recommendation for health policy makers on significant determinants to appropriate infant feeding practice. Now reads as “**Strengthening the provisions of nutritional education, integrating maternal mental health with routine maternal health care service, providing economic support to mothers with low income and health educating for multiparous women”**

**Line 48:** the vowel **“a”** is added to correct the grammar

**Result section**

**Line 209:** We made some adjustment on result section of body of manuscript. Now the sentence reads “**the proportion of infants with birth order above three was 12.7%.** To align with other statements on the result part we made change without changing the previous result report interpretation. For instance, birth order Four and above does not lose its meaning if it changed with birth order above three. Subsequently, we categorize the birth order of infants into two categories without losing its meaning.

| Birth order | |  |  |  |  |  | 0.925 |
| --- | --- | --- | --- | --- | --- | --- | --- |
| <=3 | 149 | 87.1 | 299 | 87.4 | 448 | 87.3 |  |
| >3 | 22 | 12.3 | 43 | 12.6 | 65 | 12.7 |  |

**Table 1:** maternal age p-value font changed

**Table 1:** birth order p-value has added with p-value 0.925

**Line 235:** we have added the word **“appropriate”** to the magnitude of infant feeding practice. now reads as **“Magnitude of appropriate Infant feeding practice”**

**On discussion section:** we have changed the references order to make sure the paragraphs are coherent. the reference numbers 27 become 25, reference number 25 become 26 and reference number 26 become 27. in this case no new paragraph was simply we want to have logically coherent paragraph. The paragraph continued with **“However, an inverse relationship was found in a report from northern Ghana………between postpartum depression and feeding practice”.**

**Line 301:** we correct the grammatical word and added the word **“was”** and **“appropriate”.** Now the statement reads as **“The current study determined that** **monthly income was significantly associated with appropriate infant feeding practice”**

**Line 303:** the word “**those”** was removed instead “mothers” was added to clarify the statement. Now read as **“appropriate infant feeding than mothers from less monthly income”**

**Line 309**: added the word “**due to”** to correct the grammar**.** now reads as **“**The possible reason could be **due to** the mothers with higher birth order…”.

**On limitation of the study**:

after we revise the manuscript have added and changed some words to make the sentences clear grammatically.

**Line 318 ->** the word “unable” is changed. The modified sentence read “**The limitations of the study is the possible inability to assess causality/temporal relationship between postpartum depression and feeding practices for children younger than one year”**

**Line 321->** the word “information” is added to minimize ambiguity for readers. The corrected sentence is **“However, the knowledge acquired from this research enabled us to describe the baseline information for future comparisons and/or discussions”**

Journal Requirements:

**Comment 2:** Please review your reference list to ensure that it is complete and correct. If you have cited papers that have been retracted, please include the rationale for doing so in the manuscript text, or remove these references and replace them with relevant current references. Any changes to the reference list should be mentioned in the rebuttal letter that accompanies your revised manuscript. If you need to cite a retracted article, indicate the article’s retracted status in the References list and also include a citation and full reference for the retraction notice.

**Author response:** Thank you for your constructive comments and suggestions. We took some time and checked the reference list and made some adjustments to make sure the bibliography is full and current to meet the journal requirements. None of the papers cited in the manuscript has retracted but only references number 8 has erratum and cited as per Plose One referencing style for original article with erratum.

**Reference 1:** the reference has been modified accordingly.

Kumar D, Goel NK, Mittal PC, Misra P. Influence of Infant-feeding Practices on Nutritional Status of Under-five Children. Indian J Pediatr. 2006;73(5):417–22.

Instead of

Dinesh Kumar, N.K. Goel PCM and PM. Influence of Infant-feeding Practices on Nutritional Status of Under-five Children. Indian J Pediatr. 2006;73:417–22.

**Reference 2:** the internet source reference has been cited properly. “World Health Organization (2016). Infant and young child feeding [internet]. [cited 14 Dec 2018]. Available from: <https://www.who.int/news-room/fact-sheets/detail/infant-and-young-child-feeding>.

Instead of

World Health Organization. Infant and young child feeding [internet]. Available from: https://www.who.int/news-room/fact-sheets/detail/infant-and-young-child-feeding2016.

**Reference 3:** reference 3 has been revised as follows

World Health Organization, UNICEF (2003). Global Strategy for Infant and Young Child Feeding. Geneva: World Health Organization

**Reference 5:** reference 5 has been revised accordingly and cited as

“O'Hara MW. Postpartum Depression : What We Know. J Clin Psychol. 2009;65(12):1258–69”

**Reference 6:** Should be written properly and DOI has been added.

Fisher J, Cabral de Mello M, Patel V, Rahman A, Tran T, Holton S ,et al. Prevalence and determinants of common perinatal mental disorders in women in low- and lower-middle-income countries : a systematic review. Bull World Health Organ. 2012;90(2):139–49. doi:10.2471/BLT.11.091850

**Reference 7:** We corrected according to Plose One referencing style formatting. The authors were more than six so we added et.al at the end. Now cited as

“Atuhaire C, Brennaman L, Cumber SN, Rukundo GZ, Nambozi G. The magnitude of postpartum depression among mothers in Africa: a literature review. Pan Afr Med J. 2020;37:89. doi:10.11604/pamj.2020.37.89.23572”

**Reference 8**: This source of reference has an error in **Figure 1** and corrected by women’s health (lond) journal. Therefore, to make sure the current bibliography we have included the erratum with proper erratum paper citation.

Slomian J, Honvo G, Emonts P, Reginster J, Bruyère O. Consequences of maternal postpartum depression : A systematic review of maternal and infant outcomes. Women’s Health. 2019;15:1–55. Figure 1.Erratum in: Womens Health (Lond). 2019;15:1745506519844044. doi:10.1177/1745506519844044

**Reference 9:** reference 9 should be written properly as follows

“Thorsteinsson EB, Loi NM, Farr K. Changes in stigma and help-seeking in relation to postpartum depression: non-clinical parenting intervention sample. Peer J 2018 Nov 8;6: e5893. doi:10.7717/peerj.5893”

**Reference 10**: reference 10 should be written properly as follows

Ingram J, Johnson D, Copeland M, Churchill C, Taylor H. The development of a new breast feeding assessment tool and the relationship with breast feeding self-efficacy. *Midwifery*. 2015;31(1):132-137.

**Reference 11:** : reference 11 should be written properly as follows

“Adedinsewo DA, Fleming AS, Steiner M, Meaney MJ, Girard AW; MAVAN team. Maternal anxiety and breastfeeding: findings from the MAVAN (Maternal Adversity, Vulnerability and Neurodevelopment) Study. J Hum Lact. 2014;30(1):102-109”.

**Reference 12:** reference 12 written properly as follows

“Dennis CL, McQueen K. The relationship between infant-feeding outcomes and postpartum depression: a qualitative systematic review. *Pediatrics*. 2009;123(4):e736-e751. doi:10.1542/peds.2008-1629

**Reference 13:** reference 13 written properly as follows

“O'Brien M, Buikstra E, Hegney D. The influence of psychological factors on breastfeeding duration. J Adv Nurs. 2008;63(4):397-408”.

**Reference 14:** reference 14 has been modified properly.

“Deribachew H, Berhe D, Zaid T, Desta S. Assessment of prevalence and associated factors of postpartum depression among postpartum mothers in eastern zone of Tigray. Eur J Pharm Med Res. 2016;3(10):54–60”

Instead of

Mariam D, Dessalegn B, Tadesse Z, Siyoum D. Assessment of prevalence and associated factors of postpartum depression among postpartum mothers in eastern zone of Tigray. Eur J Pharm Med Res. 2016;3(10):54–60

**Reference 15:** reference 15 has been modified properly.

“Mulugeta A, Hagos F, Kruseman G, Linderhof V, Stoecker B, Abraha Z, et al. Child malnutrition in Tigray, northern Ethiopia. East Afr Med J. 2010;87(6):248-254. doi:10.4314/eamj.v87i6.63083

**Reference 16:** reference 16 has been modified properly

Tashakori A, Behbahani AZ, Irani RD. Comparison Of Prevalence Of Postpartum Depression Symptoms Between Breastfeeding Mothers And Non-breastfeeding Mothers. Iran J Psychiatry. 2012;7(2):61-65.

**Reference 17**: The citation is changed to the preferred citation by the organization and added its internet source. Cited as follows

“Wellstart International(1996). Tool Kit for Monitoring and Evaluating Breastfeeding Practices and Programs. Available from: www.ennonline.net/bfmandetoolkit”

**References 18:** We have added the organizational author. Part 2—Measurement. Geneva, Switzerland: World Health Organization. Now the reference is cited as

**“**World Health Organization. Indicators for Assessing Infant and Young Child Feeding Practices: Part 2—Measurement. Geneva, Switzerland: World Health Organization; 2010”

**References 19:** reference 19 has been modified properly

“Hanlon C, Medhin G, Alem A, Araya M, Abdulahi A, Hughes M, et al. Detecting perinatal common mental disorders in Ethiopia : Validation of the self-reporting questionnaire and Edinburgh Postnatal Depression Scale. J Affect Disord. 2008;108(3):251–62”.

**Refrences 20:** reference 20 has been modified properly and cited as it appears below

“van der Westhuizen C, Wyatt G, Williams JK, Stein DJ, Sorsdahl K. Validation of the Self Reporting Questionnaire 20-Item (SRQ-20) for Use in a Low- and Middle-Income Country Emergency Centre Setting. Int J Ment Health Addict. 2016;14(1):37-48. doi:10.1007/s11469-015-9566-x”

**References 21:** reference 21 has been modified properly and cited as it appears below

Medhin G, Hanlon C, Dewey M, Alem A, Tesfaye F, Lakew Z, et al. The effect of maternal common mental disorders on infant undernutrition in Butajira , Ethiopia : The P-MaMiE study. BMC Psychiatry 10, 32 (2010). https://doi.org/10.1186/1471-244X-10-32

**Reference 22:** reference 22 has been modified properly and cited as it appears below

“Parsons CE, Young KS, Rochat TJ, Kringelbach ML, Stein A. Postnatal depression and its effects on child development: a review of evidence from low- and middle-income countries. Br Med Bull. 2012;101:57-79. doi:10.1093/bmb/ldr047”.

**Reference 23:** reference 23 has been modified properly and cited as it appears below

“Hasselmann MH, Werneck GL, Silva CV. Symptoms of postpartum depression and early interruption of exclusive breastfeeding in the first two months of life. Cad Saude Publica. 2008;24 suppl 2:s341–s352.”

**Reference 24:** reference 24 has been modified properly and cited as it appears below

“Madeghe BA, Kimani VN, Vander Stoep A, Nicodimos S, Kumar M. Postpartum depression and infant feeding practices in a low income urban settlement in Nairobi-Kenya. BMC Res Notes. 2016;9(1):506. Published 2016 Dec 8. doi:10.1186/s13104-016-2307-9”

**Reference 25**: reference 25 has been modified properly and cited as it appears below

“Wemakor A, Iddrisu H. Maternal depression does not affect complementary feeding indicators or stunting status of young children (6-23 months) in Northern Ghana. BMC Res Notes. 2018;11(1):408. Published 2018 Jun 25. doi:10.1186/s13104-018-3528-x”

**References 27:** reference 27 has been updated accordingly and cited as it appears below

“Areja A, Yohannes D, Yohannis M. Determinants of appropriate complementary feeding practice among mothers having children 6-23 months of age in rural Damot sore district, Southern Ethiopia. BMC Nutr. 2017;3:82. doi:10.1186/s40795-017-0202-y”.

[Note: HTML markup is below. Please do not edit.]

Reviewers' comments:

Reviewer's Responses to Questions

Comments to the Author

1. If the authors have adequately addressed your comments raised in a previous round of review and you feel that this manuscript is now acceptable for publication, you may indicate that here to bypass the “Comments to the Author” section, enter your conflict of interest statement in the “Confidential to Editor” section, and submit your "Accept" recommendation.

Reviewer #1: (No Response)

Reviewer #2: All comments have been addressed

2. Is the manuscript technically sound, and do the data support the conclusions?

The manuscript must describe a technically sound piece of scientific research with data that supports the conclusions. Experiments must have been conducted rigorously, with appropriate controls, replication, and sample sizes. The conclusions must be drawn appropriately based on the data presented.

Reviewer #1: Yes

Reviewer #2: Yes

3. Has the statistical analysis been performed appropriately and rigorously?

Reviewer #1: Yes

Reviewer #2: Yes

4. Have the authors made all data underlying the findings in their manuscript fully available?

The PLOS Data policy requires authors to make all data underlying the findings described in their manuscript fully available without restriction, with rare exception (please refer to the Data Availability Statement in the manuscript PDF file). The data should be provided as part of the manuscript or its supporting information, or deposited to a public repository. For example, in addition to summary statistics, the data points behind means, medians and variance measures should be available. If there are restrictions on publicly sharing data—e.g. participant privacy or use of data from a third party—those must be specified.

Reviewer #1: Yes

Reviewer #2: Yes

5. Is the manuscript presented in an intelligible fashion and written in standard English?

PLOS ONE does not copyedit accepted manuscripts, so the language in submitted articles must be clear, correct, and unambiguous. Any typographical or grammatical errors should be corrected at revision, so please note any specific errors here.

Reviewer #1: Yes

Reviewer #2: Yes

6. Review Comments to the Author

Please use the space provided to explain your answers to the questions above. You may also include additional comments for the author, including concerns about dual publication, research ethics, or publication ethics. (Please upload your review as an attachment if it exceeds 20,000 characters)

Reviewer #1: Thank you for addressing the reviewer feedback. Please revise the language in your conclusion from the abstract. There seems to be some confusion

This study showed low prevalence of appropriate infant feeding practice and significantly higher proportion of mother without postpartum depression practice appropriate infant feeding than their counterpart.

please add some language at the end about the low (overall) prevalence of appropriate infant feeding practices. While it is significantly lower in mothers with PPD, 42% is also very low in mothers without PPD. Please include that in your policy recommendations.

**Reviewer 1 comments:**

**Author response**: Thank you for your interesting insight, we have carefully revised and correct the statement as follows. This statement was rephrased **“The overall prevalence of appropriate infant feeding practice in the study area was low. The prevalence of appropriate infant feeding practice was significantly different between mother without postpartum depression and mother with postpartum depression”.**

**instead of**

This study showed low prevalence of appropriate infant feeding practice and significantly higher proportion of mother without postpartum depression practice appropriate infant feeding than their counterpart.

**Author response:** We have included the word **“overall”** the overall prevalence of appropriate infant feeding practices was low.

**Authors response:** Thanks for your deep insight and we have made some recommendation for strengthening nutritional education to all mothers as appropriate infant feeding is still low in women without PPD. We added this recommendation to policy makers and it was very helpful. “Therefore, the modified statement reads as “**strengthening the provisions of nutritional education, providing economic support to mothers with low income, health educating for multiparous and integrating maternal mental health with maternal health care services**”**.** in addition, we recommend the policy makers to consider women with low income and birth order above three as it mentioned in the above phrase.

Reviewer #2: I consider the revision adequate and the results interesting to be published. The subject is simple but relevant to mother and child health, therefore my advice would be to publish it.

7. PLOS authors have the option to publish the peer review history of their article (what does this mean?). If published, this will include your full peer review and any attached files.

If you choose “no”, your identity will remain anonymous but your review may still be made public.

Do you want your identity to be public for this peer review? For information about this choice, including consent withdrawal, please see our Privacy Policy.

Reviewer #1: No

Reviewer #2: No

[NOTE: If reviewer comments were submitted as an attachment file, they will be attached to this email and accessible via the submission site. Please log into your account, locate the manuscript record, and check for the action link "View Attachments". If this link does not appear, there are no attachment files.]

While revising your submission, please upload your figure files to the Preflight Analysis and Conversion Engine (PACE) digital diagnostic tool, https://pacev2.apexcovantage.com/. PACE helps ensure that figures meet PLOS requirements. To use PACE, you must first register as a user. Registration is free. Then, login and navigate to the UPLOAD tab, where you will find detailed instructions on how to use the tool. If you encounter any issues or have any questions when using PACE, please email PLOS at figures@plos.org. Please note that Supporting Information files do not need this step.
