# Supplementary material for: The relationship between postpartum depression and appropriate infant feeding practice in eastern zone of Tigray, Ethiopia: A comparative cross-sectional study
Source: PLoS One. 2023 Jan 25;18(1):e0280141. doi: 10.1371/journal.pone.0280141 (PMC9876352; doi:10.1371/journal.pone.0280141)
Supplement: Attachment — Submitted filename: Response to Reviewers.docx [file pone.0280141.s007.docx]

Dear Editors,

We truly appreciate the time and effort put forth by editors to improve our manuscript, ‘‘The relationship between postpartum depression and appropriate infant feeding practice in Eastern zone of Tigray, Ethiopia: A comparative cross-sectional study”. We have revised and incorporated the changes to the manuscript to address all the insightful suggestions raised by the editors.

PONE-D-21-02467R2

THE RELATIONSHIP BETWEEN POSTPARTUM DEPRESSION AND APPROPRIATE INFANT FEEDING PRACTICE IN EASTERN ZONE OF TIGRAY, ETHIOPIA: A COMPARATIVE CROSS-SECTIONAL STUDY

PLOS ONE

Dear Dr. Weldu,
Thank you for submitting your manuscript to PLOS ONE. After careful consideration, we feel that it has merit but does not fully meet PLOS ONE’s publication criteria as it currently stands. Therefore, we invite you to submit a revised version of the manuscript that addresses the points raised during the review process.

Few minor corrections required. In abstract, please clearly define appropriate infant feeding practices in methods.  Include results for income in abstract results.

Please submit your revised manuscript by May 20 2022 11:59PM. If you will need more time than this to complete your revisions, please reply to this message or contact the journal office at [plosone@plos.org](mailto:plosone@plos.org). When you're ready to submit your revision, log on to <https://www.editorialmanager.com/pone/> and select the 'Submissions Needing Revision' folder to locate your manuscript file.
Please include the following items when submitting your revised manuscript:

- A rebuttal letter that responds to each point raised by the academic editor and reviewer(s). You should upload this letter as a separate file labeled 'Response to Reviewers'.
- A marked-up copy of your manuscript that highlights changes made to the original version. You should upload this as a separate file labeled 'Revised Manuscript with Track Changes'.
- An unmarked version of your revised paper without tracked changes. You should upload this as a separate file labeled 'Manuscript'.

If you would like to make changes to your financial disclosure, please include your updated statement in your cover letter. Guidelines for resubmitting your figure files are available below the reviewer comments at the end of this letter.

If applicable, we recommend that you deposit your laboratory protocolsin [protocols.io](http://protocols.io/) to enhance the reproducibility of your results. Protocols.io assigns your protocol its own identifier (DOI) so that it can be cited independently in the future. For instructions see: <https://journals.plos.org/plosone/s/submission-guidelines#loc-laboratory-protocols>. Additionally, PLOS ONE offers an option for publishing peer-reviewed Lab Protocol articles, which describe protocols hosted on [protocols.io](http://protocols.io/). Read more information on sharing protocols at <https://plos.org/protocols?utm_medium=editorial-email&utm_source=authorletters&utm_campaign=protocols>.

We look forward to receiving your revised manuscript.

Kind regards,

Melissa F. Young, Ph.D.
Academic Editor
PLOS ONE

Journal Requirements:

Please review your reference list to ensure that it is complete and correct. If you have cited papers that have been retracted, please include the rationale for doing so in the manuscript text, or remove these references and replace them with relevant current references. Any changes to the reference list should be mentioned in the rebuttal letter that accompanies your revised manuscript. If you need to cite a retracted article, indicate the article’s retracted status in the References list and also include a citation and full reference for the retraction notice.

[Note: HTML markup is below. Please do not edit.]

Reviewers' comments:

[NOTE: If reviewer comments were submitted as an attachment file, they will be attached to this email and accessible via the submission site. Please log into your account, locate the manuscript record, and check for the action link "View Attachments". If this link does not appear, there are no attachment files.]

While revising your submission, please upload your figure files to the Preflight Analysis and Conversion Engine (PACE) digital diagnostic tool, <https://pacev2.apexcovantage.com/>. PACE helps ensure that figures meet PLOS requirements. To use PACE, you must first register as a user. Registration is free. Then, login and navigate to the UPLOAD tab, where you will find detailed instructions on how to use the tool. If you encounter any issues or have any questions when using PACE, please email PLOS at [figures@plos.org](mailto:figures@plos.org). Please note that Supporting Information files do not need this step.

**Editor’s comment**: Few minor corrections required. In abstract, please clearly define appropriate infant feeding practices in methods. Include results for income in abstract results.

**Author response:** Thank you for your insightful comments.

**In the methods part of the abstract,** we have changed infant feeding practice into appropriate infant feeding practice and restated as “Appropriate infant feeding practice was assessed using structured questionnaire obtained from Monitoring and Evaluating Breastfeeding Practices toolkit.

**In the result section of the abstract,** we have added household monthly income as follows “Households with monthly income 1000-1999 ETB (AOR= 2.26; 95% CI: 1.01-5.08), 2000-2999 ETB (AOR= 1.96; 95% CI: 1.21-4.73) and 3000-3999 ETB (AOR= 5.13; 95% CI: 1.97-13.4) were more likely to practice appropriate infant feeding”
