# Supplementary material for: The relationship between postpartum depression and appropriate infant feeding practice in eastern zone of Tigray, Ethiopia: A comparative cross-sectional study
Source: PLoS One. 2023 Jan 25;18(1):e0280141. doi: 10.1371/journal.pone.0280141 (PMC9876352; doi:10.1371/journal.pone.0280141)
Supplement: Attachment — Submitted filename: Responses to Reviewers.docx [file pone.0280141.s008.docx]

Dear Editors,

We greatly appreciate the time and effort put forth by reviewers and editors to improve our paper, ‘‘The relationship between postpartum depression and appropriate infant feeding practice in Eastern zone of Tigray, Ethiopia: A comparative cross-sectional study”. We have revised and incorporated the changes in the manuscript to address all the insightful suggestions raised by the editors and the reviewers.

PONE-D-21-02467R3

THE RELATIONSHIP BETWEEN POSTPARTUM DEPRESSION AND APPROPRIATE INFANT FEEDING PRACTICE IN EASTERN ZONE OF TIGRAY, ETHIOPIA: A COMPARATIVE CROSS-SECTIONAL STUDY

PLOS ONE

Dear Dr. Weldu,

Thank you for submitting your manuscript to PLOS ONE. After careful consideration, we feel that it has merit but does not fully meet PLOS ONE’s publication criteria as it currently stands. Therefore, we invite you to submit a revised version of the manuscript that addresses the points raised during the review process.

Please submit your revised manuscript by Sep 24 2022 11:59PM. If you will need more time than this to complete your revisions, please reply to this message or contact the journal office at plosone@plos.org. When you're ready to submit your revision, log on to https://www.editorialmanager.com/pone/ and select the 'Submissions Needing Revision' folder to locate your manuscript file.

Please include the following items when submitting your revised manuscript:

A rebuttal letter that responds to each point raised by the academic editor and reviewer(s). You should upload this letter as a separate file labeled 'Response to Reviewers'.

A marked-up copy of your manuscript that highlights changes made to the original version. You should upload this as a separate file labeled 'Revised Manuscript with Track Changes'.

An unmarked version of your revised paper without tracked changes. You should upload this as a separate file labeled 'Manuscript'.

If you would like to make changes to your financial disclosure, please include your updated statement in your cover letter. Guidelines for resubmitting your figure files are available below the reviewer comments at the end of this letter.

If applicable, we recommend that you deposit your laboratory protocols in protocols.io to enhance the reproducibility of your results. Protocols.io assigns your protocol its own identifier (DOI) so that it can be cited independently in the future. For instructions see: https://journals.plos.org/plosone/s/submission-guidelines#loc-laboratory-protocols. Additionally, PLOS ONE offers an option for publishing peer-reviewed Lab Protocol articles, which describe protocols hosted on protocols.io. Read more information on sharing protocols at https://plos.org/protocols?utm_medium=editorial-email&utm_source=authorletters&utm_campaign=protocols.

We look forward to receiving your revised manuscript.

Kind regards,

Melissa F. Young, Ph.D.

Academic Editor

PLOS ONE

Journal Requirements:

Please review your reference list to ensure that it is complete and correct. If you have cited papers that have been retracted, please include the rationale for doing so in the manuscript text, or remove these references and replace them with relevant current references. Any changes to the reference list should be mentioned in the rebuttal letter that accompanies your revised manuscript. If you need to cite a retracted article, indicate the article’s retracted status in the References list and also include a citation and full reference for the retraction notice.

Please adjust the reviewers' comments.

[Note: HTML markup is below. Please do not edit.]

Reviewers' comments:

Reviewer's Responses to Questions

Comments to the Author

1. If the authors have adequately addressed your comments raised in a previous round of review and you feel that this manuscript is now acceptable for publication, you may indicate that here to bypass the “Comments to the Author” section, enter your conflict of interest statement in the “Confidential to Editor” section, and submit your "Accept" recommendation.

Reviewer #1: All comments have been addressed

Reviewer #3: (No Response)

2. Is the manuscript technically sound, and do the data support the conclusions?

The manuscript must describe a technically sound piece of scientific research with data that supports the conclusions. Experiments must have been conducted rigorously, with appropriate controls, replication, and sample sizes. The conclusions must be drawn appropriately based on the data presented.

Reviewer #1: (No Response)

Reviewer #3: Yes

3. Has the statistical analysis been performed appropriately and rigorously?

Reviewer #1: (No Response)

Reviewer #3: I Don't Know

4. Have the authors made all data underlying the findings in their manuscript fully available?

The PLOS Data policy requires authors to make all data underlying the findings described in their manuscript fully available without restriction, with rare exception (please refer to the Data Availability Statement in the manuscript PDF file). The data should be provided as part of the manuscript or its supporting information, or deposited to a public repository. For example, in addition to summary statistics, the data points behind means, medians and variance measures should be available. If there are restrictions on publicly sharing data—e.g. participant privacy or use of data from a third party—those must be specified.

Reviewer #1: (No Response)

Reviewer #3: Yes

5. Is the manuscript presented in an intelligible fashion and written in standard English?

PLOS ONE does not copyedit accepted manuscripts, so the language in submitted articles must be clear, correct, and unambiguous. Any typographical or grammatical errors should be corrected at revision, so please note any specific errors here.

Reviewer #1: (No Response)

Reviewer #3: No

6. Review Comments to the Author

Please use the space provided to explain your answers to the questions above. You may also include additional comments for the author, including concerns about dual publication, research ethics, or publication ethics. (Please upload your review as an attachment if it exceeds 20,000 characters)

Reviewer #1: (No Response)

Reviewer #3: (No Response)

**Editor’s comment**

**Comment 1:** Any changes to the reference list should be mentioned in the rebuttal letter that accompanies your revised manuscript.

**Author response:** Thank you, our paper has largely improved after providing another revision. We added more references to provide proper context about proper infant feeding practices and removed unnecessary details from the discussion part. The main purpose of including the references is to explain the magnitude of appropriate infant feeding in the study area to the reader and to provide a glimpse into feeding practice in Ethiopia. These are references that have been added to help explain the outcome variables.

21. Shagaro SS, Mulugeta BT, Kale TD. Complementary feeding practices and associated factors among mothers of children aged 6-23 months in Ethiopia: Secondary data analysis of Ethiopian mini demographic and health survey 2019. Arch Public Heal. 2021;79(1):1–12.

22. Demilew YM, Tafere TE, Abitew DB. Infant and young child feeding practice among mothers with 0 – 24 months old children in Slum areas of Bahir Dar City, Ethiopia. Int Breastfeed J. 2017;12:26:1–9.

23. Kassa T, Meshesha B, Haji Y, Ebrahim J. Appropriate complementary feeding practices and associated factors among mothers of children age 6-23 months in. BMC Pediatr. 2016;16(131):1-10.

24. Fanta M, Cherie HA. Magnitude and determinants of appropriate complementary feeding practice among mothers of children age 6–23 months in Western Ethiopia. PLoS ONE 2020;15(12):e0244277. http://dx.doi.org/10.1371/journal.pone.0244277

25. Yonas F, Asnakew M, Wondafrash M, Abdulahi M. Infant and Young Child Feeding Practice Status and Associated Factors among Mothers of under 24-Month-Old Children in Shashemene Woreda, Oromia Region, Ethiopia. Open Access Libr J. 2015;2,1-15.

26. Mekonnen M, Kinati T, Bekele K, Tesfa B, Hailu D, Jemal K. Infant and young child feeding practice among mothers of children age 6 to 23 months in Debrelibanos district, North Showa zone, Oromia region, Ethiopia. PLoS ONE. 2021;16(9):e0257758.

27. Hassen SL, Temesgen MM, Marefiaw TA, Ayalew BS, Abebe DD, Desalegn SA. Infant and Young Child Feeding Practice Status and Its Determinants in Kalu District, Northeast Ethiopia: Community-Based Cross-Sectional Study. Nutr Diet Suppl. 2021;13:67–81.

**Response to the comments**

2. Is the manuscript technically sound, and do the data support the conclusions?

**Reviewer #1:** (No Response)

**Author response:** Thank you for your suggestion. To ensure reliable information was collected the principal investigator provides proper training for data collectors, and then supervisors check the data accuracy and completeness. In addition, the tools used to measure the outcome were based on WHO core indicators for appropriate infant feeding practice. We have Clearly defined all outcomes (appropriate infant feeding practice) and exposures (postpartum depression). The measurement for infant feeding practice and postpartum depression was contextualized and checked for reliability (internal consistency). Moreover, we have ensured the data comparability between postpartum depression and non-depressed mothers by at least taking participants of control from the same area and checking whether there is a significant difference in sociodemographic factors between postpartum depressed and non-depressed mothers. Thus, the information helped us to achieve the purpose of the study, which is to assess the magnitude of appropriate infant feeding practice and to determine the effect of postpartum depression on infant feeding practice. So, our conclusion is in line with the objective of the study.

3. Has the statistical analysis been performed appropriately and rigorously?

Reviewer #1: (No Response)

Reviewer #3: I Don't Know

**Author response:** Thank you for your concern. During analysis, we tried to control potential confounders using multivariable regression. multicollinearity was checked for all the independent variables that are included in the model that had VIF (variance inflation factor) of less than 1.8 and tolerance greater than 0.52. The data also checked for outliers in continuous variables, none of them were significant after being diagnosed by COOKs distance, and the assumptions of binary logistic regression were checked. In addition, interaction among independent variables was assessed during binary logistic regression, and Hosmer and Lemeshow test was also carried out to check model fitness which was not significant (p-value = 0.95). Then, variables with p<=0.25 in the bivariate analysis were included in the final model to consider public health and clinically important variables. Finally, backward logistic regression was used to determine independent predictors of appropriate infant feeding. The sample size was adequate with a high response rate (97%).

4. Have the authors made all data underlying the findings in their manuscript fully available?

Reviewer #1: (No Response)

**Author response:** all the necessary data has been uploaded as a supporting information file.

5. Is the manuscript presented in an intelligible fashion and written in standard English?

PLOS ONE does not copyedit accepted manuscripts, so the language in submitted articles must be clear, correct, and unambiguous. Any typographical or grammatical errors should be corrected at revision, so please note any specific errors here.

Reviewer #1: (No Response)

Reviewer #3: No

**Author response:** Thank you, we have made a significant correction after carefully reviewing the manuscript's entire text for typographical and grammatical errors using Grammarly software. In addition, the font size of the table text has been changed to standard.

6. Review Comments to the Author

Please use the space provided to explain your answers to the questions above. You may also include additional comments for the author, including concerns about dual publication, research ethics, or publication ethics. (Please upload your review as an attachment if it exceeds 20,000 characters)

Reviewer #1: (No Response)

Reviewer #3: (No Response)

**Author response:** none of the findings of the manuscript has been attempted to publish in other journals.

7. PLOS authors have the option to publish the peer review history of their article (what does this mean?). If published, this will include your full peer review and any attached files.

If you choose “no”, your identity will remain anonymous but your review may still be made public.

Do you want your identity to be public for this peer review? For information about this choice, including consent withdrawal, please see our Privacy Policy.

Reviewer #1: No

Reviewer #3: No

[NOTE: If reviewer comments were submitted as an attachment file, they will be attached to this email and accessible via the submission site. Please log into your account, locate the manuscript record, and check for the action link "View Attachments". If this link does not appear, there are no attachment files.]

While revising your submission, please upload your figure files to the Preflight Analysis and Conversion Engine (PACE) digital diagnostic tool, https://pacev2.apexcovantage.com/. PACE helps ensure that figures meet PLOS requirements. To use PACE, you must first register as a user. Registration is free. Then, login and navigate to the UPLOAD tab, where you will find detailed instructions on how to use the tool. If you encounter any issues or have any questions when using PACE, please email PLOS at figures@plos.org. Please note that Supporting Information files do not need this step.
