# Supplementary material for: The relationship between postpartum depression and appropriate infant feeding practice in eastern zone of Tigray, Ethiopia: A comparative cross-sectional study
Source: PLoS One. 2023 Jan 25;18(1):e0280141. doi: 10.1371/journal.pone.0280141 (PMC9876352; doi:10.1371/journal.pone.0280141)
Supplement: Attachment — Submitted filename: Response to Reviewers.docx [file pone.0280141.s009.docx]

Dear Editors,

We truly appreciate the time and effort put forth by editors to improve our manuscript, ‘‘The relationship between postpartum depression and appropriate infant feeding practice in Eastern zone of Tigray, Ethiopia: A comparative cross-sectional study”. We have revised and incorporated the changes to the manuscript to address all the insightful suggestions raised by the editors.

PONE-D-21-02467R4

THE RELATIONSHIP BETWEEN POSTPARTUM DEPRESSION AND APPROPRIATE INFANT FEEDING PRACTICE IN EASTERN ZONE OF TIGRAY, ETHIOPIA: A COMPARATIVE CROSS-SECTIONAL STUDY

PLOS ONE

Dear Dr. Weldu,

Thank you for submitting your manuscript to PLOS ONE. After careful consideration, we feel that it has merit but does not fully meet PLOS ONE’s publication criteria as it currently stands. Therefore, we invite you to submit a revised version of the manuscript that addresses the points raised during the review process.

ACADEMIC EDITOR:

Please include infant age in weeks as a variable in table 1.

for your analysis of appropriate infant feeding practices please separate the sample into <6 months and 6 -12 months since the definition of appropriate feeding is different for these age groups

please add infant age in weeks as a variable in table 3.

for S1 Figure please separate out by age <6 months and 6-12 months

**Response to Academic editor:**

**Editor’s comment:** Please include infant age in weeks as a variable in table 1

**Authors response:** Thank you for the comment, we have included infant age in months. In the descriptive statistics, (line 209) we have converted the mean age of the infants from days to months and added infant age in table 1. Converting infant age from days to weeks brings some information to be missed while categorizing as less than six months (0-26 weeks) and six and above months (26.1-52.14 weeks), so to overcome this, we used infant age in months as the main objective of this research is not to understand which age specifically associated with appropriate infant feeding.

Moreover, we used infant age in months, to ensure comparability of this study with others as almost all previously conducted similar researches have categorized infant age in months.

**Editor’s comment**: for your analysis of appropriate infant feeding practices please separate the sample into <6 months and 6 -12 months since the definition of appropriate feeding is different for these age groups.

**Authors response:** we have done the analysis separately for the two age groups, below 6 month and 6- 11 months. Consequently, two new tables have been added.

**Editors comment:** please add infant age in weeks as a variable in table 3.

**Authors response:** Thank you for the comment, we have added infant age to Table 5 (previously labeled as Table 3). This brings some changes to the values and interpretation of the findings, including the AOR of the variables of the final step of backward LR. For instance, the odds of appropriate infant feeding practice were 1.71 times higher among mothers without postpartum depression compared to their counterparts (AOR = 1.71; 95% CI: 1.08-2.69). This statement from the result part was changed to " The odds of appropriate infant feeding practice were 2 times higher among mothers without postpartum depression compared to their counterparts (AOR = 2.03; 95% CI: 1.29-3.18)." due to the addition of infant age as a variable in Table 5.

In addition, the effect of birth order was also changed from “The odds of appropriate infant feeding practices among infants with a birth order above three was 48% (AOR = 0.52; 95% CI: 0.28-0.97) less than those infants with a birth order of three and below” to “The odds of appropriate infant feeding practices among infants with a birth order above three was 58% (AOR = 0.42; 95% CI: 0.26-0.97) less than those infants with a birth order of three and below”.

Corresponding to these changes, in the abstract section, similar changes was made.

**Editors comment**: for S1 Figure please separate out by age <6 months and 6-12 months

**Authors response:** Thank you for the comment, we have separately represented appropriate infant feeding among the age groups, 6 months and 6-11 months, using a bar graph.

Please ensure that your decision is justified on PLOS ONE’s publication criteria and not, for example, on novelty or perceived impact.

For Lab, Study and Registered Report Protocols: These article types are not expected to include results but may include pilot data.

Please submit your revised manuscript by Dec 03 2022 11:59PM. If you will need more time than this to complete your revisions, please reply to this message or contact the journal office at plosone@plos.org. When you're ready to submit your revision, log on to https://www.editorialmanager.com/pone/ and select the 'Submissions Needing Revision' folder to locate your manuscript file.

Please include the following items when submitting your revised manuscript:

A rebuttal letter that responds to each point raised by the academic editor and reviewer(s). You should upload this letter as a separate file labeled 'Response to Reviewers'.

A marked-up copy of your manuscript that highlights changes made to the original version. You should upload this as a separate file labeled 'Revised Manuscript with Track Changes'.

An unmarked version of your revised paper without tracked changes. You should upload this as a separate file labeled 'Manuscript'.

If you would like to make changes to your financial disclosure, please include your updated statement in your cover letter. Guidelines for resubmitting your figure files are available below the reviewer comments at the end of this letter.

If applicable, we recommend that you deposit your laboratory protocols in protocols.io to enhance the reproducibility of your results. Protocols.io assigns your protocol its own identifier (DOI) so that it can be cited independently in the future. For instructions see: https://journals.plos.org/plosone/s/submission-guidelines#loc-laboratory-protocols. Additionally, PLOS ONE offers an option for publishing peer-reviewed Lab Protocol articles, which describe protocols hosted on protocols.io. Read more information on sharing protocols at https://plos.org/protocols?utm_medium=editorial-email&utm_source=authorletters&utm_campaign=protocols.

We look forward to receiving your revised manuscript.

Kind regards,

Tanya Doherty, PhD

Academic Editor

PLOS ONE

Journal Requirements:

Please review your reference list to ensure that it is complete and correct. If you have cited papers that have been retracted, please include the rationale for doing so in the manuscript text, or remove these references and replace them with relevant current references. Any changes to the reference list should be mentioned in the rebuttal letter that accompanies your revised manuscript. If you need to cite a retracted article, indicate the article’s retracted status in the References list and also include a citation and full reference for the retraction notice.

[Note: HTML markup is below. Please do not edit.]

[NOTE: If reviewer comments were submitted as an attachment file, they will be attached to this email and accessible via the submission site. Please log into your account, locate the manuscript record, and check for the action link "View Attachments". If this link does not appear, there are no attachment files.]

While revising your submission, please upload your figure files to the Preflight Analysis and Conversion Engine (PACE) digital diagnostic tool, https://pacev2.apexcovantage.com/. PACE helps ensure that figures meet PLOS requirements. To use PACE, you must first register as a user. Registration is free. Then, login and navigate to the UPLOAD tab, where you will find detailed instructions on how to use the tool. If you encounter any issues or have any questions when using PACE, please email PLOS at figures@plos.org. Please note that Supporting Information files do not need this step.
